# Isolation, identification, and sensitivity profile of *Bacillus spp*. in co-infection with respiratory viruses

**DOI:** 10.3389/fped.2025.1718515

**Published:** 2026-01-27

**Authors:** Angelica de Lima das Chagas, Joilma Cruz da Silva Araújo, Jose Daniel Goncalves Vieira, Melissa Amelotti Gomes Avelino, Lilian Carla Carneiro

**Affiliations:** 1 Health Science Post Graduation, Medical College, Federal University of Goias, Goiania, Brazil; 2 Pharmacy Faculty, Federal University of Goias, Goiania, Brazil; 3 Postgraduate Studies in Biology of the Host-Parasite Relationship, Federal University of Goias, Goiania, Brazil

**Keywords:** bacterial coinfection, antibiogram, *Bacillus spp*. resistance, respiratory virus, COVID-19

## Abstract

This study investigates the occurrence and antimicrobial susceptibility of *Bacillus spp*. in pediatric patients with viral respiratory infections admitted to intensive care units. Secondary bacterial infections are known to exacerbate the severity of viral respiratory diseases and represent a major cause of morbidity and mortality during pandemics, including COVID-19. A total of 659 respiratory samples from children with respiratory symptoms hospitalized in five hospitals were analyzed. Bacterial co-infections were identified by inoculation in BHI medium and confirmed by MALDI-TOF. Antimicrobial susceptibility testing was performed using the Kirby-Bauer method, following EUCAST guidelines. Among 166 cases of bacterial co-infection, 20 (12.05%) were attributed to *Bacillus spp*., with a predominance in patients infected with respiratory syncytial virus (55%). The isolates showed high susceptibility to vancomycin (85%), imipenem (80%), erythromycin (70%), and ciprofloxacin (65%). These findings reveal that *Bacillus spp*., often considered an environmental contaminant, may play a clinically relevant role in pediatric viral co-infections, particularly in severe respiratory cases. This study contributes novel data to a poorly explored area of pediatric infectious disease research, emphasizing the need for routine susceptibility testing to optimize antimicrobial therapy. The results provide a foundation for future molecular studies on *Bacillus spp*. virulence and resistance mechanisms, supporting evidence-based management and infection control practices in critical care settings.

## 1 Introduction

Human respiratory viruses include a wide range of viral agents that infect cells of the respiratory tract, cause respiratory symptoms, and are transmitted primarily through the respiratory secretions of infected individuals. Infections caused by respiratory viruses are often indistinguishable clinically. These viruses belong to several viral families, with variations in their structures and genomes, populations susceptible to infection, severity of disease, seasonality of circulation, transmissibility, and modes of transmission (1).

Viral presence can make the respiratory niche more prone to bacterial colonization by several mechanisms. First, viruses can make the epithelium more susceptible to bacterial colonization by altering mucosal surfaces. This can result in damage to the cilia, decreasing the effectiveness of mucociliary clearance in the respiratory epithelium. Furthermore, damage caused by viruses and deterioration of the integrity of the epithelial lining can facilitate bacterial colonization and subsequent translocation. Virus-infected cells can reduce the production of antimicrobial peptides, such as β-defensins, thereby compromising the epithelium's natural host defense capabilities. Viral neuraminidase (NA) activity can expose bacterial receptors by cleaving sialic acid residues that are hiding them. Finally, viruses can promote both bacterial colonization and replication, either directly or indirectly (2).

Respiratory viruses that can predispose to such secondary infections include respiratory syncytial virus, measles virus, parainfluenza virus, adenovirus, rhinovirus, and, more recently, the COVID-19 virus. In the case of the COVID-19 virus, the emergence of this co-infection simultaneously with COVID-19 not only adds a layer of complexity to the disease but may also trigger new forms of tissue pathology, increasing the challenges faced by healthcare professionals in diagnosing and effectively treating the disease (3, 4).

Among human respiratory viruses, respiratory syncytial virus (RSV) is one of the main agents, capable of triggering lower respiratory tract infections in infants and young children and is the main causative agent of bronchiolitis. The complexity of respiratory viral infections is even more pronounced during pandemics, such as the recent COVID-19 pandemic, which has had an impact on a global scale. RSV, along with other respiratory viruses such as rhinovirus, has been detected in cases of respiratory infections, contributing to the complexity of clinical diagnosis during the COVID-19 pandemic. These viruses share similar symptoms to COVID-19, which may lead to mistaken suspicion of the disease in cases of infection by other viral agents (5).

The domestication of animals and urban concentration contributed to close contact between humans, animals, and the environment, leading to the emergence of diseases of zoonotic origin (6). The COVID-19 pandemic, triggered by the emergence of the novel coronavirus SARS-CoV-2, has become one of the most significant events of the 21st century. The rapid spread of the virus and its ability to transmit efficiently between humans have caused an unprecedented global health crisis (7, 8).

The etiological agent of COVID-19 was named severe acute respiratory syndrome coronavirus 2 (SARS-CoV-2) on March 11, 2020, by the World Health Organization (WHO) (9). The symptoms of the disease, in milder cases, cough, sore throat, fever are some of the most common symptoms; in more severe cases, the clinical manifestations are dyspnea followed by respiratory discomfort, persistent pressure, oxygen saturation less than 95%, especially in elderly patients, with comorbidities and often the infection can be asymptomatic (10, 11).

Transmission between people occurs through common routes, such as direct contact, physical contact, and airborne transmission via aerosols, especially during medical interventions. Coughing, sneezing, inhalation of droplets, and contact with mucous membranes of the mouth, nose, and eyes are the main means of spreading the virus (12). Furthermore, the spread of SARS-CoV-2 is exacerbated by its long incubation period, which typically extends to about 5–6 days and can range from 0 to as long as 24 days. What is even more challenging is that asymptomatic, pre-symptomatic, or mildly symptomatic individuals can spread the virus without being aware of it, making control of the pandemic more difficult (13, 14).

In the study by Yasir et al., (15), it was possible to identify the presence of bacterial co-infections associated with viral infections. The study analyzed a total of 107 swabs, 22 from control individuals and 85 from patients with COVID-19. Through RT-PCR assays, the researchers identified several respiratory pathogens responsible for these secondary infections. In addition to *Bacillus spp*., significant amounts of other bacterial genera were observed, such as *Sphingomonas spp., Kurthia spp., Microbacterium spp., Methylobacterium spp., Brevibacillus spp., Acinetobacter spp., Lactococcus spp.* and *Haemophilus spp*. Known pathogens such as *Staphylococcus aureus*, *Streptococcus pneumoniae, Haemophilus influenzae, Moraxella catarrhalis, Salmonella spp.,* and *Klebsiella pneumoniae*. These findings highlight the complexity of co-infections, reinforcing the importance of considering the presence of bacteria in patients with COVID-19.

The genus *Bacillus spp*. is extensive, with over 543 species, and includes both pathogenic and nonpathogenic strains. For example*, B. anthracis* is recognized as pathogenic, being the causative agent of anthrax, a serious disease. On the other hand, *B. cereus* is associated with cases of food poisoning and can become pathogenic in certain circumstances. Meanwhile, *B. thuringiensis* is often considered nonpathogenic to humans, being used as a biopesticide to control insects, although it can pose risks to people with compromised immune systems (16–18).

Bacillus cereus is rarely associated with lower respiratory tract infections. In the study conducted by Shimoyama *et al*. (19), A case of pneumonia caused by *B. cereus* was described in an 81-year-old immunocompetent female patient with heart problems. She had a cough, difficulty breathing, swelling in her legs, fever, and blood in her stool. Tests revealed fluid accumulation in her lungs and congestion. After treatment, she improved, but on the second day, respiratory complications developed. Blood tests detected the presence of *B. cereus*. On the fourth day, the situation worsened, resulting in serious respiratory problems and pneumonia. The patient was transferred to the ICU and treated with a combination of antibiotics, including imipenem and levofloxacin, which proved effective. After treatment, she recovered and was discharged from the ICU after a few days.

The endospores of *Bacillus spp*. confer antimicrobial resistance, which contributes to the spread of these organisms. Generally found in nature and not linked to a specific host, bacteria of the genus *Bacillus spp*. are widely distributed, interacting with humans through different pathways, such as soil, air, plants, and even the human gastrointestinal tract (20).

Antibiotics have been extensively used in hospital settings, especially in intensive care units, to prevent or treat potential secondary infections. Analysis finds that about half of ICU patients with SARS-CoV-2 infection were treated with antibiotics, with little attention given to bacterial co-infection (21). This highlights the unnecessary and excessive use of these antimicrobials in mild and moderate cases of COVID-19, potentially increasing the risks of adverse events and the emergence of multidrug-resistant bacterial pathogens (22).

According to Lefrançois and collaborators (23), to deal with emergencies such as the recent pandemic, it is essential to consider the environmental, social, economic, ethical, and political factors that characterize a social ecosystem and influence the emergence of zoonoses. The response to infectious disease emergencies needs to be comprehensive to facilitate the identification and rapid control of zoonotic risks before they affect humans, and it is also necessary to establish an effective health surveillance system capable of providing early and reliable information on new pathogens. The aim of this study was to identify and evaluate the antimicrobial sensitivity profile of *Bacillus spp*. in co-infection with respiratory viruses in children in ICUs.

## 2 Methodology

This research is an experimental and epidemiological study, approved by the ethics committee at CEP/HC under protocol number: CAAE: 33540320.7.0000.5078.

All biological material came from the biorepository of the project entitled: “Differential diagnosis and pediatric clinical evolution of COVID-19 in the context of seasonality of respiratory viruses in a capital city in the Midwest of Brazil”. The study cited is an investigation of the incidence of viral types that make up the viral panel, including the COVID-19 virus (which is not part of the viral panel, but caused a pandemic).

Samples of secretion from the nasopharynx of children (≤ 14 years old) admitted to the following hospitals in Goiás: Materno Infantil, Hospital da Criança, Hospital de Doenças trópicais and Hugol were included in the present study. Records on demographic characteristics (age and sex), patient symptoms and comorbidities were collected. All patients had some respiratory symptoms, influenza, pneumonia, suspected COVID-19 and confirmed cases of COVID-19. Nasopharyngeal swab samples were collected and stored in viral transport medium (MTV) and kept frozen at −80 °C. The MTV medium contained the antimicrobials gentamicin and amphotericin B.

### 2.1 Bacterial isolation

Samples were recovered in brain-heart infusion (BHI) broth, where 200 μL of the sample were pipetted into 3 mL of BHI medium. The samples were then incubated for 24 to 48 h in an orbital shaker, maintained at a temperature of 37 °C and a shaking speed of 150.2 rpm, favoring bacterial growth. Turbidity analysis was performed to verify bacterial growth, which was considered positive when turbidity formation was observed in the culture medium. Samples positive for BHI were inoculated using the composite streak method on nutrient agar for 24 h (Plastlabor, Brazil). Isolated colonies were selected for MALD-TOF. The entire bacterial isolation process was performed in triplicate, to confirm true isolates and not environmental contaminants.

### 2.2 Identification by MALDI-TOF

The isolates were identified by matrix-assisted laser desorption/ionization mass spectrometry and time-of-flight (MALDI-TOF MS®) using the BrükerMicroflex LT/SH instrument (Bruker Daltonics GmbH & Co. KG, Bremen, Germany®), with a score value > 2.0.

### 2.3 Antimicrobial susceptibility testing

The analysis was performed using the Kirby-Bauer disk diffusion method. Initially, colonies identified by MALD-TOF as Bacillus spp. were cultured for 24 h and transferred to test tubes containing sterile 0.85% saline solution and then homogenized by shaking. The resulting solutions were standardized according to the McFarland scale to achieve a concentration of 0.5 and then seeded onto Petri dishes containing Mueller-Hinton agar. After seeding, discs impregnated with antimicrobials, including meropenem (10 µg), ciprofloxacin (5 µg), vancomycin (5 µg), erythromycin (15 µg), and linezolid (10 µg), were seeded onto Mueller-Hinton agar, which were then incubated at 35 °C for 24 h. Next, the inhibition halos formed around each disc were measured using calipers, and the values obtained were compared with the cut-off points established by BrCAST.

## 3 Results

A total of 659 pediatric patients were included in this study., with 78% being male and 22% female. MALDI-TOF mass spectrometry identified 20 isolates belonging to the genus Bacillus, as shown in Supplementary Table S1, which describes the genus, species, and identification log obtained by MALDI-TOF. Among these patients, 166 cases of bacterial co-infection were identified (Table 1), and 20 (12.05%) of these co-infections were caused by *Bacillus spp*.

**Table 1:** Microorganisms species found in patients with respiratory viruses, with the corresponding percentages of occurrence.

Bacterial species	Quantity found (%)
*Enterococus faecalis*	74 (44.6%)
*Bacillus spp.*	20 (12.05%)
*Staphylococcus spp.*	18 (10.84%)
*Xanthomanas*	1 (0.6%)
*Microbacterium hominis*	3 (1.81%)
*Escherichia coli*	5 (3.01%)
*Acinetobacter radioresistents*	2 (1.2%)
*Corynebacterium sanguinis*	1 (0.6%)
*Peribacillus muralis*	1 (0.6%)
*Priestia megaterium*	1 (0.6%)
*Enterobacter hormaechi*	10 (6.02%)
Fungal species	Quantity found (%)
*Candida parapsilosis*	22 (13.25%)
*Meyerozyma guilliermondii*	8 (4.82%)

Author's own.

### 3.1 Clinical characteristics and virus identified

The most common symptoms on admission were fever and cough, followed by dyspnea and irritability (Table 2). Only 3 patients had comorbidities, namely heart disease, neurogenic bladder, osteosarcoma, and mediastinal tumor. Bacterial co-infection by *Bacillus spp*. occurred in greater numbers in patients with respiratory syncytial virus (RSV) (11.55%). Of the patients, 6 (30%) received antibiotics and antivirals, while 1 (5%) received only antivirals and another 1 (5%) received only antibiotics. Furthermore, in 12 (60%) cases, no specific treatment was administered.

**Table 2:** Viruses associated with co-infection, symptoms and medications used in patients with Bacillus spp.

Virus found	Symptoms and comorbidities	Medications used
Respiratory syncytial virus (RSV)	Flu, cough, fever >37.8, dyspnea, tachypnea, O2 catheter for four days	Tamiflu antiviral, clavulin antibiotic
Severe acute respiratory syndrome (SARS), pneumonia (PNM) and sepsis	Fever >37.8	Antiviral Tamiflu, antibiotic ceftriaxone and oxacillin
Respiratory syncytial virus (RSV)	Respiratory distress, fever >37.8, cough, dyspnea, tachypnea, loss of appetite	Antiviral oseltamivir, antibiotic ampicillin + gentamicin
Respiratory syncytial virus (RSV)	Respiratory distress, cough, nasal obstruction, dyspnea, tachypnea, irritability	Antiviral oseltamivir, antibiotic clarithromycin
Respiratory syncytial virus (RSV)	Respiratory distress, cough, nasal obstruction, dyspnea, tachypnea, irritability	Antiviral oseltamivir, antibiotic clarithromycin
Acute respiratory infection (ARI)	Heart disease, fever >37.8, dyspnea	Ceftriaxone antibiotic
COVID-19	fever >37.8	Antiviral oseltamivir
Respiratory syncytial virus (RSV)	Coryza, abdominal pain, nasal obstruction, rhinorrhea, constipation, oliguria. Carrier of hydrocephalus and neurogenic bladder	Antiviral oseltamivir, antibiotic ceftriaxone
Lung involvement 20%, respiratory distress	Fever >37.8, cough, sneezing, rhinorrhea, dyspnea, tachypnea	–
Respiratory syncytial virus type A (RSV), Bronchiolitis	–	–
Respiratory syncytial virus type B (RSV)	Severe asthma attack	–
COVID-19, Bronchiolitis, Respiratory syncytial virus type A (RSV)	–	–
Bronchopulmonary dysplasia (BDP), bronchiolitis and pneumonia (PNM)	–	–
Cystic fibrosis	–	–
Flu	Osteosarcoma and Mediastinal Tumor	–
Bronchiolitis, Respiratory syncytial virus type B (RSV)	–	–
Rhinovirus, Bronchiolitis		–
Rinovírus	Asthma attack	–
Respiratory syncytial virus (RSV)	–	–
Severe acute respiratory syndrome (SARS), pneumonia (PNM)	–	–

Author's own.

### 3.2 Antimicrobial susceptibility patterns of bacterial isolates

It was observed that *Bacillus spp*. strains showed resistance to antimicrobial agents of different classes. Most strains were susceptible to vancomycin (17/20 85%), followed by imipenem (16/20 80%), erythromycin (14/20 70%), ciprofloxacin (13/20 65%) and 85% were resistant to linezolid (17/20).

## 4 Discussion

The MALDI-TOF results revealed the presence of bacterial co-infection in 166 patients with respiratory viruses, with 12.05% of these co-infections caused by *Bacillus spp*. This high incidence, especially associated with *Bacillus spp*., highlights the complex interaction between viruses and bacteria during viral respiratory infections.

Bacterial co-infection with *Bacillus spp*. in our study was observed in a variety of clinical contexts, with a higher incidence in patients with respiratory syncytial virus, representing 11 (55%) of the cases. Additionally, cases of co-infection were identified in patients with several conditions, such as SARS, PNM, COVID-19, cystic fibrosis, influenza virus, bronchiolitis, acute respiratory infection, and Rhinovirus. Unusual situations were also observed, such as one patient presenting co-infection with COVID-19, bronchioliti,s and RSV, in addition to two patients with SARS and pneumonia simultaneously.

The most common symptoms at admission of patients included in our study were fever and cough, followed by dyspnea, irritability, tachypnea, loss of appetite, nasal obstruction, respiratory distress, runny nose, abdominal pain, rhinorrhea, constipation, sneezing, and, in isolated cases, asthma attacks and the need for an oxygen catheter for four days. Three patients had comorbidities, including heart disease, neurogenic bladder, osteosarcoma, and mediastinal tumor.

This finding is in line with the research of Yasir et al., (15), which identified occurrences of bacterial co-infections associated with SARS-CoV-2, including a significant presence of the genus *Bacillus spp*. in nasopharyngeal swab samples. Both this study and ours highlighted that symptoms such as fever and cough were the most frequently reported among the analyzed COVID-19 patients, underlining the complexity of bacterial co-infections during the viral disease.

Similarly Zhan et al. (24), identified *Bacillus spp*. as one of the dominant bacteria in patients with lung infections in their study. This finding highlights the relevance of *Bacillus spp*. in respiratory infection settings. Regarding antimicrobial treatment, the drugs used to treat the patients in this study were ceftriaxone, clavulin, oxacillin, ampicillin + gentamicin, and clarithromycin. Each of these drugs has distinct mechanisms of action that target different components of the bacterial cell wall or metabolic processes, offering broad coverage against a variety of pathogens. After treatment, all patients demonstrated satisfactory recovery and were discharged from the hospital, suggesting a positive response to the treatments administered.

The choice of these classes of antimicrobials may have been based on criteria such as efficacy, safety, and broad spectrum of action, as well as the ability to cover pneumococcal, gram-negative, and atypical bacterial infections in the pulmonary context. It is important to note that the treatment was empirical, since there was no confirmation of bacterial co-infection through testing, as recommended by the Brazilian Guidelines for Pharmacological Treatment of COVID-19, which advise against the inappropriate use of broad-spectrum antibacterials and the prescription of prophylactic antibiotics in patients with suspected or diagnosed COVID-19 in the absence of confirmed bacterial co-infection (24).

Patients with mild symptoms may receive antibiotics for community-acquired pneumonia, such as amoxicillin, azithromycin, or fluoroquinolones (levofloxacin, moxifloxacin, ciprofloxacin), while those with severe symptoms should be treated with an empiric approach that covers all possible pathogens (25, 26).

Regarding the antimicrobial susceptibility test, of the 20 isolates studied, 17 (85%), 16 (80%), 14 (70%), and 13 (65%) were susceptible to vancomycin, imipenem, erythromycin, and ciprofloxacin, respectively. Therefore, the most indicated drug for the treatment of bacterial coinfection by *Bacillus spp*. is vancomycin, due to its demonstrated high sensitivity. Vancomycin belongs to the glycopeptide class and exhibits activity against several Gram-positive aerobic and anaerobic microorganisms. Its mechanism of action involves binding to the peptidoglycan of the bacterial cell wall, a heteropolymer composed of long chains of polysaccharides and peptides (27). Additional studies, such as that by Fahim *et al*. (28), demonstrated the high susceptibility of vancomycin in 30 isolates of Bacillus species using the minimum inhibitory concentration (MIC) test.

Furthermore, as stated by Celandroni *et al*. (29), In their study, all *Bacillus spp*. species were found to be susceptible to vancomycin and ciprofloxacin, as determined by the minimum inhibitory concentration (MIC). It is crucial to highlight the importance of antimicrobial susceptibility testing, such as the minimum inhibitory concentration (MIC) test, to detect these resistance patterns. These tests provide accurate information about the sensitivity or resistance of a given bacterium to antibiotics, allowing physicians to select the most appropriate treatment for the patient. Using antibiotics without considering bacterial resistance can lead to treatment failures, prolonged illness, increased healthcare costs, and the development of even more widespread antibiotic resistance (30).

This study highlights the incidence of secondary co-infections by *Bacillus spp*. together with several respiratory viruses during the pandemic period. It is worth mentioning that both studies on co-infection by *Bacillus spp*. associated with viral infections and antimicrobial susceptibility testing for *Bacillus spp*. are limited, with a shortage of similar studies. This gap made it difficult to compare with other studies, highlighting the urgent need for more attention and research in this specific area.

## 5 Conclusion

*Bacillus spp*., although historically linked to soil, is also detected in hospitalized patients, revealing their ability to act as pathogens or opportunists in humans. This adaptability shows that Bacillus bacteria can easily adapt to new environments and cause infections in vulnerable people, thanks to their genetic and metabolic flexibility. The high incidence of bacterial co-infections with *Bacillus spp*., especially during viral respiratory infections such as COVID-19 and other severe respiratory conditions, underscores the clinical relevance of these microbiological interactions.

Our research also identified varied clinical symptoms among affected patients, including fever, cough, and dyspnea, reflecting the diversity of clinical presentations associated with *Bacillus spp*. and respiratory virus co-infections. These findings reinforce the need for accurate diagnostic and therapeutic strategies to address these complex pathogenic interactions.

Furthermore, the efficacy of vancomycin against *Bacillus spp*., as evidenced by antimicrobial susceptibility testing, underscores the importance of appropriately selecting antibiotic treatments based on accurate bacterial susceptibility data. The observed resistance to certain antimicrobial agents, such as linezolid, also highlights the continued need for surveillance and research to understand and address these microbiological challenges.

Therefore, continued research on *Bacillus spp*. in clinical settings is essential to improve the management of complex infections and to inform public health policies that promote the rational use of antibiotics and the prevention of antimicrobial resistance.

In addition to filling gaps in scientific literature, our study will provide valuable insights into the co-infection of *Bacillus spp*. with respiratory viruses, an area that is still underexplored. The results obtained will not only be essential to guide future research, such as molecular identification and genetic profiling of the pathogens involved but may also provide a solid basis for the appropriate choice of antimicrobials in these cases of co-infection. This is essential to improve the clinical management of patients co-infected by *Bacillus spp*. associated with respiratory viruses, promoting more favorable health outcomes and supporting effective infection control strategies.

## Data Availability

The raw data supporting the conclusions of this article will be made available by the authors, without undue reservation.

## References

[1] Leung NHL. Transmissibility and transmission of respiratory viruses. Nat Rev Microbiol. (2021) 19:528–45. 10.1038/s41579-021-00535-6 33753932 PMC7982882

[2] Bosch AATM Biesbroek G Trzcinski K Sanders EAM Bogaert D. Viral and bacterial interactions in the upper respiratory tract. PLoS Pathog. (2013) 9:e1003057. 10.1371/journal.ppat.1003057 23326226 PMC3542149

[3] McCullers JA. Insights into the interaction between influenza virus and pneumococcus. Clin Microbiol Rev. (2006) 19:571–82. 10.1128/CMR.00058-05 16847087 PMC1539103

[4] Patton MJ Gaggar A Might M Erdmann N Orihuela CJ Harrod KS. Community-acquired bacterial coinfections and COVID-19. Physiol Rev. (2024) 104:1–21. 10.1152/physrev.00010.2023 37589392

[5] Calderaro A De Conto F Buttrini M Piccolo G Montecchini S Maccari C Human respiratory viruses, including SARS-CoV-2, circulating in the winter season 2019–2020 in Parma, northern Italy. Int J Infect Dis. (2021) 102:79–84. 10.1016/j.ijid.2020.09.1473 33017694 PMC7530558

[6] Maia L Bonilha Dubugras MT. Reflexões sobre a pandemia de COVID-19 na perspectiva da Saúde Única. Instituto de Saúde. (2023). Available online at: https://hdl.handle.net/11324/22103 (Accessed April 1, 2025).

[7] Chakraborty I Maity P. COVID-19 outbreak: migration, effects on society, global environment and prevention. Sci Total Environ. (2020) 728:138882. 10.1016/j.scitotenv.2020.138882 32335410 PMC7175860

[8] Ali SA Baloch M Ahmed N Ali AA Iqbal A. The outbreak of coronavirus disease 2019 (COVID-19)—an emerging global health threat. J Infect Public Health. (2020) 13:644–6. 10.1016/j.jiph.2020.02.033 32199792 PMC7102664

[9] WHO. Coronavirus disease (COVID-19) pandemic. (2020) Available online at: https://www.who.int/europe/emergencies/situations/covid-19 (Accessed April 1, 2025).

[10] Liao Q-Q Zhu Z-F Zhu K-W Yang Z Liu G-L Li X-Q Symptoms can predict COVID-19 pneumonia in patients infected with SARS-CoV-2 omicron variants. Sci Rep. (2024) 14:30037. 10.1038/s41598-024-81156-w 39627318 PMC11615324

[11] Wu C Chen X Cai Y Xia J Zhou X Xu S Risk factors associated with acute respiratory distress syndrome and death in patients with coronavirus disease 2019 pneumonia in Wuhan, China. JAMA Intern Med. (2020) 180:934–43. 10.1001/jamainternmed.2020.0994 32167524 PMC7070509

[12] Lv J Gao J Wu B Yao M Yang Y Chai T Aerosol transmission of coronavirus and influenza virus of animal origin. Front Vet Sci. (2021) 8:572012. 10.3389/fvets.2021.572012 33928140 PMC8078102

[13] Xu X Wu Y Kummer AG Zhao Y Hu Z Wang Y Assessing changes in incubation period, serial interval, and generation time of SARS-CoV-2 variants of concern: a systematic review and meta-analysis. BMC Med. (2023) 21:374. 10.1186/s12916-023-03070-8 37775772 PMC10541713

[14] Wang Y Wang Y Chen Y Qin Q. Unique epidemiological and clinical features of the emerging 2019 novel coronavirus pneumonia (COVID-19) implicate special control measures. J Med Virol. (2020) 92:568–76. 10.1002/jmv.25748 32134116 PMC7228347

[15] Yasir M Al-Sharif HA Al-Subhi T Sindi AA Bokhary DH El-Daly MM Analysis of the nasopharyngeal microbiome and respiratory pathogens in COVID-19 patients from Saudi Arabia. J Infect Public Health. (2023) 16:680–8. 10.1016/j.jiph.2023.03.001 36934642 PMC9984237

[16] Bbosa N Ssemwanga D Weiss SL Kalungi S Mawanda A Ssentudde R Ssekyeru E Ssekagiri A Kiiza R Rwankindo C Identification of anthrax as the cause of a cluster of unexplained deaths, Uganda, 2023: the role of metagenomic next-generation sequencing and postmortem specimens. Am J Trop Med Hyg. (2025) 112(4):835–9. 10.4269/ajtmh.24-0489 39773989 PMC11965767

[17] Zhou Q Li G Cui Y Xiang J Zhu S Li S Genomic characterization of Bacillus cereus isolated from food poisoning cases revealed the mechanism of toxin production. Front Microbiol. (2024) 14:1238799. 10.3389/fmicb.2023.1238799 38282728 PMC10822677

[18] Koilybayeva M Shynykul Z Ustenova G Waleron K Jońca J Mustafina K Gas chromatography–mass spectrometry profiling of volatile metabolites produced by some Bacillus spp. and evaluation of their antibacterial and antibiotic activities. Molecules. (2023) 28:7556. 10.3390/molecules28227556 38005278 PMC10673538

[19] Shimoyama Y Umegaki O Ooi Y Agui T Kadono N Minami T. Bacillus cereus pneumonia in an immunocompetent patient: a case report. JA Clin Rep. (2017) 3:25. 10.1186/s40981-017-0096-3 29457069 PMC5804607

[20] Baindara P Aslam B. Editorial: bacillus spp. - transmission, pathogenesis, host-pathogen interaction, prevention and treatment. Front Microbiol. (2023) 14:1307723. 10.3389/fmicb.2023.1307723 37928694 PMC10622664

[21] Abu-Rub LI Abdelrahman HA Johar A-RA Alhussain HA Hadi HA Eltai NO. Antibiotics prescribing in intensive care settings during the COVID-19 era: a systematic review. Antibiotics (Basel). (2021) 10:935. 10.3390/antibiotics10080935 34438985 PMC8389042

[22] Vellano PO de Paiva MJM. O uso de antimicrobiano na COVID-19 e as infecções: o que sabemos. Res Soc Dev. (2020) 9:e841997245. 10.33448/rsd-v9i9.7245

[23] Lefrançois T Malvy D Atlani-Duault L Benamouzig D Druais P-L Yazdanpanah Y After 2 years of the COVID-19 pandemic, translating one health into action is urgent. Lancet. (2023) 401:789–94. 10.1016/S0140-6736(22)01840-2 36302392 PMC9595398

[24] Zhang T Wu Q Zhang Z. Probable pangolin origin of SARS-CoV-2 associated with the COVID-19 outbreak. Curr Biol. (2020) 30:1346–1351.e2. 10.1016/j.cub.2020.03.022 32197085 PMC7156161

[25] Falavigna M Colpani V Stein C Azevedo LCP Bagattini AM de Brito GV Guidelines for the pharmacological treatment of COVID-19. The task-force/consensus guideline of the Brazilian association of intensive care medicine, the Brazilian society of infectious diseases and the Brazilian society of pulmonology and tisiology. Rev Bras ter Intensiva. (2020) 32:166–96. 10.5935/0103-507X.20200039 32667444 PMC7405746

[26] Metlay JP Waterer GW. Treatment of community-acquired pneumonia during the coronavirus disease 2019 (COVID-19) pandemic. Ann Intern Med. (2020) 173:304–5. 10.7326/M20-2189 32379883 PMC7236892

[27] Schmid PJ Forstner P Kittinger C. Sliding motility of Bacillus cereus mediates vancomycin pseudo-resistance during antimicrobial susceptibility testing. J Antimicrob Chemother. (2024) 79:1628–36. 10.1093/jac/dkae156 38785365 PMC11215547

[28] Fahim NAE Ismail GAE-W Abdelaleem MB Elanany M Saber SM EL-Ashry MAE-R. Identification, characterization and antibiotic susceptibility testing for Bacillus species. Iran J Microbiol. (2022) 14:484–94. 10.18502/ijm.v14i4.10234 36721507 PMC9867631

[29] Celandroni F Salvetti S Gueye SA Mazzantini D Lupetti A Senesi S Identification and pathogenic potential of clinical Bacillus and Paenibacillus isolates. PLoS One. (2016) 11:e0152831. 10.1371/journal.pone.0152831 27031639 PMC4816569

[30] Lin TZ Jayasvasti I Tiraphat S Pengpid S Jayasvasti M Borriharn P. The predictors influencing the rational use of antibiotics among public sector: a community-based survey in Thailand. Drug Healthc Patient Saf. (2022) 14:27–36. 10.2147/DHPS.S339808 35369038 PMC8965102

