# Supplementary material for: Isolation, identification, and sensitivity profile of Bacillus spp. in co-infection with respiratory viruses
Source: Front Pediatr. 2026 Jan 27;13:1718515. doi: 10.3389/fped.2025.1718515 (PMC12886364; doi:10.3389/fped.2025.1718515)
Supplement: Supplementary file 1 [file Table1.docx]

Supplementary Material

# Supplementary Tables

**Table S1.** Identification of Bacillus genus isolates by MALDI-TOF mass spectrometry

| **Organism** | **Log value (score)** |
| --- | --- |
| *Bacillus amyloliquefaciens* | 1.72 |
| *Bacillus amyloliquefaciens* | 1.74 |
| *Bacillus amyloliquefaciens* | 1.77 |
| *Bacillus cereus* | 1.80 |
| *Bacillus subtilis* | 2.18 |
| *Bacillus subtilis* | 1.94 |
| *Bacillus subtilis* | 2.04 |
| *Bacillus subtilis* | 2.14 |
| *Bacillus subtilis* | 1.87 |
| *Bacillus subtilis* | 1.90 |
| *Bacillus subtilis* | 1.98 |
| *Bacillus subtilis* | 1.95 |
| *Bacillus subtilis* | 1.91 |
| *Bacillus subtilis* | 1.70 |
| *Bacillus subtilis* | 2.14 |
| *Bacillus subtilis* | 1.90 |
| *Bacillus subtilis* | 2.18 |
| *Bacillus velezensis* | 1.78 |
| *Bacillus velezensis* | 1.80 |
| *Bacillus velezensis* | 1.89 |

Source: Author's own
